# Supplementary material for: Quantification of 363 Pesticides in Leafy Vegetables (Dill, Rocket and Parsley) in the Turkey Market by Using QuEChERS with LC-MS/MS and GC-MS/MS
Source: Foods. 2023 Feb 28;12(5):1034. doi: 10.3390/foods12051034 (PMC10000932; doi:10.3390/foods12051034)
Supplement: Supplementary file 1 [file foods-12-01034-s001.zip › foods-2197622-supplementary.pdf]

Supplementary materials

Table S1. MRM parameters for 311 pesticide residues by LC-MS/MS.

Pesticide	MRM transitions	
	quantifier ion, *m*/*z* (collision energy, eV)	qualifier ion, *m*/*z* (collision energy, eV)
2,4-D	219.0/160.8 (20)	219.0/124.9 (38)
Acephate	184.0/142.9 (13)	184.0/124.9 (25)
Acetamiprid	223.1/126 (31)	223.1/99.0 (55)
Acibenzolar-s-methyl	211.0/136.1 (41)	211.0/91.1 (29)
Aldicarb	208.1/116.0 (11)	208.1/88.9 (20)
Aldicarb sulfone	223.2/86.1 (21)	223.2/147.9 (13)
Aldicarb sulfoxide	207.2/132.0 (11)	207.2/88.9 (19)
Aminocarb	209.1/152.1 (19)	209.1/137.2 (31)
Amitraz	294.0/163.0 (17)	294.0/122.0 (41)
Anilofos	368.0/199.0 (21)	368.0/125.0 (45)
Atrazine-desethyl	188.1/146.1 (23)	188.1/110.1 (31)
Atrazine-desisopropyl	174.1/104.1 (33)	174.1/132.1 (25)
Azaconazole	300.0/159.0 (37)	300.0/231.0 (23)
Azamethiphos	325.0/183.0 (23)	325.0/138.9 (37)
Azinphos ethyl	346.0/132.1 (23)	346.0(160.1 (15)
Azinphos methyl	318.0/132.0 (21)	318.0/160.0 (13)
Azoxystrobin	404.1/372.0 (21)	404.1/344.1 (35)
Benalaxyl	326.0/148.0 (27)	326.0/208.0 (21)
Benazolin	243.9/220.9 (32)	243.9/170.0 (23)
Bendiocarb	224.1/167.2 (13)	224.1/108.9 (21)
Benodanil	324.0/231.0 (13)	324.0/120.0 (25)
Benoxacor	260.1/149.1 (25)	260.1/134.1 (41)
Bensulfuron-methyl	411.1/148.9 (27)	411.1/119.0 (51)
Bentazone	239.0/131.9 (38)	239.0/196.9 (28)
Bitertanol	338.0/269.0 (15)	338.0/70.0 (25)
Bixafen	411.9/90.9 (58)	411.9/279.8 (34)
Boscalid	343.0/307.0 (27)	343.0/140.0 (27)
Bromacil	261.0/205.0 (33)	261.0/188.0 (36)
Bromoxynil	275.7/78.6 (40)	275.7/80.6 (42)
Bromuconazole	378.0/159.0 (37)	378.0/70.0 (35)
Bupirimate	317.2/166.0 (35)	317.2/108.0 (37)
Buprofezin	306.2/201.0 (19)	306.2/115.9 (23)
Butachlor	312.2/238.1 (31)	312.2/162.1 (35)
Butafenacil	492.2/331.0 (31)	492.2/180.2 (63)
Butamifos	350.0/333.1 (15)	350.0/152.0 (31)
Buturon	237.2/84.2 (23)	237.2/126.1 (41)
Butylate	218.1/57.1 (29)	218.1/156.2 (15)
Cadusafos	271.1/158.9 (21)	271.1/214.9 (13)
Carbaryl	202.1/145.0 (15)	202.1/127.0 (41)
Carbendazim	192.1/160.0 (29)	192.1/132.0 (45)
Carbofuran	222.1/165.0 (17)	222.1/123.1 (31)
Carboxin	236.0/143.0 (21)	236.0/87.0 (33)
Chlorbromuron	295.1/225.9 (27)	295.1/182.0 (23)

Chlorfluazuron	539.9/383.0 (47)	539.9/158.0 (47)
Chloridazon	222.0/92.0 (35)	222.0/104.0 (31)
Chloroxuron	291.0/72.0 (41)	291.0/218.1 (35)
Chlorpyrifos	350.0/96.9 (49)	350.0/197.9 (27)
Chlorsulfuron	358.0/141.0 (23)	358.0/167.0 (25)
Cinosulfuron	414.1/183.1 (21)	414.1/157.0 (33)
Clethodim	360.0/164.0 (25)	360.0/268.0 (17)
Clodinafop propargyl	350.1/266.0 (23)	350.1/91.2 (43)
Clofentezine	303.1/138.1 (21)	303.1/102.2 (61)
Clomazone	240.0/125.0 (27)	240.0/89.0 (65)
Clopyralid	191.9/146.0 (29)	191.9/110.1 (47)
Clothianidin	250.0/169.1 (17)	250.0/132.0 (19)
Crimidine	172.2/136.1 (29)	172.2/107.1 (37)
Crotoxyphos	315.1/211.0 (24)	315.1/193.0 (27)
Cyanazine	241.1/214.1 (25)	241.1/216.1 (25)
Cyazofamid	325.0/108.0 (19)	325.0/261.0 (15)
Cycloate	216.2/154.2 (17)	216.2/134.2 (19)
Cyclohexamide	282.2/264.0 (15)	282.2/246.1 (21)
Cycloxydim	326.2/280.1 (21)	326.2/252.1 (33)
Cycluron	199.2/111.1 (35)	199.2/89.1 (21)
Cyflufenamid	413.2/295.0 (23)	413.2/141.0 (33)
Cyhalofop-butyl	375.1/256.1 (21)	375.1/358.1 (11)
Cymoxanil	199.0/127.9 (13)	199.0/111.0 (18)
Cyproconazole	292.0/70.0 (49)	292.0/125.1 (37)
Cyprodinil	226.1/93.1 (49)	226.1/77.0 (65)
Daimuron	269.2/151.1 (15)	269.2/134.1 (19)
Demeton-s-(disulfoton oxon)	259.1/89.1 (6)	259.1/61.0 (16)
Demeton-s-methyl sulfone	263.0/168.9 (23)	263.0/120.8 (23)
Demeton-s-methyl sulfoxide	247.1/169.0 (17)	247.1/125.0 (19)
Desmedipham	318.0/136.0 (33)	318.0/182.0 (19)
Desmetryn	214.1/172.1 (5)	214.1/82,0 (7)
Diafenthiuron	385.2/329.2 (27)	385.2/278.1 (43)
Diazinon	305.1/169.0 (31)	305.1/97.0 (47)
Diazinon-oxon	289.1/261.1 (2)	289.1/233.1 (4)
Dichlormid	208,0/140.0 (27)	208,0/98.1 (31)
Dichlorprop	233.0/161.0 (5)	233/125.0 (5)
Dichlorvos	221.0/108.9 (25)	221.0/127.0 (25)
Diclobutrazol	328.1/199.0 (21)	328.1/159.0 (26)
Dicrotophos	238.0/112.0 (10)	238.0/193.0 (10)
Diethofencarb	268.1/226.1 (13)	268.1/124.1 (41)
Difenoconazole	406.0/251.0 (37)	406.0/188.0 (37)
Difenoxuron	287.1/214.1 (5)	287.1/214.1 (5)
Diflubenzuron	310.9/158.0 (6)	310.9/141.0 (15)
Dimefuron	339.1/295.1 (7)	339.1/256.0 (18)
Dimethachlor	256.4/224.3 (8)	256.4/148.3 (24)
Dimethenamid	276.1/244.1 (7)	276.1/168.2 (21)
Dimethoate	230.0/198.9 (3)	230.0/125 (17)
Dimethomorph	388.2/301 (17)	388.2/165 (31)
Dimetilan	241.0/196.1 (4)	241.0/72 (14)

Dimoxystrobin	327.1/205.1 (12)	327.1/116.1 (23)
Diniconazole	326.2/70.2 (25)	326.2/43.2 (47)
Dinitramine	323.0/289.0 (14)	323.0/261.0 (12)
Dinocap	382.1/86.0 (2)	382.1/69 (4)
Dinotefuran	203.1/157.1 (21)	203.1/129.1 (21)
Dioxacarb	224.0/167.1 (2)	224.0/123.0 (12)
Diphenamid	240.3/167.1 (19)	240.3/134.2 (17)
Disulfoton sulfone	306.9/171.0 (8)	206.9/153.1 (8)
Disulfoton sulfoxide	290.9/184.9 (8)	290.9/157 (20)
Dithianon	295.9/263.8 (12)	295.9/237.9 (12)
Dithiopyr	402.1/382.1 (3)	402.1/360.0 (3)
Diuron	233.1/72.2 (17)	233.1/46.3 (13)
Dodine	228.2/71.1 (20)	228.2/57.1 (22)
Edifenphos	311.0/283.0 (12)	311.0/220.0 (16)
Epoxiconazole	330.1/121.1 (17)	330.1/100.9 (40)
Eprinomectin	914.5/186.1 (23)	914.5/154.1 (23)
Esprocarb	266.2/196.1 (6)	266.2/142.1 (12)
Etaconazole	330.0/161 (12)	330.0/159 (12)
Ethametsulfuron-methyl	411.0/196.0 (12)	411.0/168.0 (34)
Ethidimuron	265.0/208.0 (11)	265.0/162.0 (15)
Ethiofencarb	226.1/164.1 (3)	226.1/107.1 (9)
Ethiofencarb sulfone	258.1/201.1 (10)	258.1/107.1 (20)
Ethiofencarb sulfoxide	242.1/185.1 (5)	242.1/107.1 (15)
Ethiolate	162.1/134.1 (11)	162.1/100.1 (14)
Ethion	385.0/199.0 (3)	385.0/143.0 (21)
Ethimirol	210.1/140.2 (21)	210.1/98.0 (27)
Ethofumesate	287.1/259.1 (3)	287.1/121.1 (11)
Ethoprophos	243.0/172.9 (10)	243.0/130.9 (20)
Etofenprox	394.1/359.1 (2)	394.1/177.0 (6)
Etoxazole	360.0/141.0 (15)	360.0/113.0 (23)
Famoxadone	392.1/331.1 (2)	392.1/238.1 (10)
Fenamidone	312.1/236.1 (9)	312.1/92.1 (20)
Fenamiphos	304.1/234 (11)	304.1/217.0 (19)
Fenamiphos sulfone	233.1/125.0 (20)	233.1/161.0 (20)
Fenamiphos sulfoxide	262.1/220.1 (18)	262.1/131.1 (31)
Fenazaquin	307.2/161.1 (11)	307.2/125.0 (31)
Fenbuconazole	337.1/125.0 (31)	337.1/70.1 (17)
Fenhexamid	302.0/97.0 (20)	302.0/55.0 (35)
Fenobucarb	208.1/152.1 (5)	208.1/95.1 (10)
Fenathiocarb	254.0/72.0 (14)	254.0/160.0 (8)
Fenoxanil	329.1/302.1 (3)	329.1/189.1 (6)
Fenoxaprop-ethyl	362.1/288.0 (15)	362.1/244.1 (12)
Fenoxycarb	302.1/287.9 (20)	302.1/88.0 (11)
Fenpropathrin	350.1/97.0 (34)	350.1/125.0 (14)
Fenpropidin	274.2/147.1 (30)	274.2/117.0 (50)
Fenpropimorph	304.4/147.1 (29)	304.1/98.2 (29)
Fenpyrazamine	332.1/304.1 (13)	332.1/290.1 (13)
Fenpyroximate	422.2/138.1 (32)	422.1/366.1 (15)
Fensulfothion	309.1/281.0 (9)	309.1/253.0 (13)

Fenthion	279.0/247.0 (5)	279.0/169.0 (11)
Fenthion oxon	262.9/231.0 (12)	262.9/215.9 (24)
Fenthion oxonsulfone	295.1/217.1 (14)	295.1/104.1 (20)
Fenthion oxonsulfoxide	279.1/264.1 (14)	279.1/104.1 (26)
Fenthion sulfone	311.0/279.9 (14)	311.0/125.0 (28)
Fenthion sulfoxide	295.0/280.0 /14)	295.0/109.0 (32)
Fenuron	165.1/120.0 (3)	165.1/95.0 (5)
Fipronil	434.7/398.8 (4)	434.7/329.8 (10)
Flamprop isopropyl	364.1/105.0 (29)	364.1/77.0 (73)
Flamprop-methyl	336.1/105.0 (14)	336.1/95.0 (20)
Florasulam	360.4/192.1 (10)	360.4/129.2 (24)
Fluazifop	456.0/223.0 (15)	456.0/344.0 (10)
Fluazifop-p-butyl	384.2/328.1 (32)	384.2/282.1 (20)
Fluazinam	462.9/415.9 (13)	462.9/397.9 (9)
Flubendiamide	681.0/277.9 /24)	681.0/253.9 (40)
Fludioxonil	247.0/180.1 (22)	247.0/126.0 (24)
Flufenoxuron	489.1/141.1 (30)	489.1/158.1 (15)
Flumetsulam	326.0/262.1 (13)	326.0/192.0 (23)
Fluometuron	233.3/160.1 (24)	233.3/72.4 (20)
Fluopyram	397.0/207.8 (22)	397.0/172.9 (32)
Fluoxastrobin	459.4/427.4 (14)	459.4/188.3 (36)
Fluridone	330.1/310.1 (2)	330.1/309.1 (2)
Flurochloridone	311.9/291.9 (16.0)	311.9/53.0 (30)
Flutolanil	324.0/262.0 (20)	324.0/242.0 (20)
Flutriafol	231.2/61.3 (27)	231.2/89.2 (10)
Fluxapyroxad	382.0/362.0 (10)	382.0/342.0 (20)
Fomesafen	456.1/344.0 (21)	456.1/222.9 (45)
Forchlorfenuron	248.2/155.1 (10)	248.2/129.2 (14)
Formothion	250.0/143.0 (6)	250.0/258.0 (2)
Furalaxyl	302.0/242.0 (15)	302.0/95.0 (25)
Furathiocarb	383.2/252.0 (7)	383.2/195.0 (13)
Haloxyfop-etotyl	434.0/316.0 (15)	434.0/73.1 (13)
Haloxyfop-methyl	376.0/316.1 (32)	376.0/288.0 (25)
Heptenophos	251.0/125.0 (15)	251.0/109.0 (30)
Hexaconazole	314.1/159.0 (31)	314.1/70.1 (17)
Hexaflumuron	461.0/158.0 (10)	461.0/141.0 (20)
Hexazinone	253.2/171.1 (11)	253.2/71.2 (31)
Hexythiazox	353.1/271.0 (6)	353.1/228.0 (8)
Imazalil	297.1/159.0 (19)	297.1/41.2 (31)
Imazapyr	262.0/217.0 (18)	262.0/148.9 (26)
Imazaquin	312.0/199.0 (29)	312.0/128.0 (50)
Imibenconazole	410.9/170.8 (18)	410.9/125.0 (34)
Imidacloprid	256.1/209.0 (10)	256.1/175.0 (12)
Indanofan	341.1/323.1 (0)	341.1/295.1 (3)
Indoxacarb	528.1/203.0 (36)	528.1/150.0 (16)
Iodosulfuron-methyl sodium	508.0/167.0 (17)	508.0/141.0 (35)
Ioxynil	369.8/215.0 (36)	369.8/127.0 (30)
Ipconazole	334.0/125.0 (48)	334.0/70.0 (30)
Iprobenfos	289.0/205.1 (4)	289.0/91.0 (20)

Isocarbamid	186.1/130.1 (7)	186.1/87.1 (11)
Isoprocarb	211.5/137.1 (13)	211.5/95.0 (22)
Isopropalin	310.2/276.2 (2)	310.2/226.1 (4)
Isopyrazam	360.2/340.2 (20)	360.2/320.2 (24)
Isoxaben	333.0/165.0 (17)	333.0/107.0 (58)
Isoxathion	314.1/170.0 (19)	314.1/105.0 (23)
Kresoxim-methyl	314.2/267.1 (3)	314.2/222.1 (7)
Lactofen	479.1/344.0 (0)	479.1/223.0 (2)
Lenacil	235.2/153.0 (11)	235.2/136.0 (33)
Linuron	249.1/182.0 (11)	249.1/160.0 (13)
Lufenuron	510.9/158.0 (16)	510.9/141.0 (44)
Malaoxon	315.1/127 (10)	315.1/99 (20)
Malathion	330.9/285.0 (0)	330.9/127.0 (4)
MCPA	201.0/142.9 (10)	201.0/140.9 (10)
Mecarbam	330.1 /226.9 (5)	330.1/198.9 (10)
Mefenacet	299.1/192.0 (3)	299.1/148.1 (3)
Mepanipyrim	224.0/106.0 (26)	224.0/77.1 (46)
Mepronil	270.0/228.0 (14)	270.0/119.0 (16)
Mesosulfuron-methyl	502.1/346.9 (20)	502.1/266.8 (34)
Metaflumizone	505.0/302.0 (10)	505.0/117.0 (48)
Metalaxyl-M	280.2/220.2 (7)	280.2/192.1 (13)
Metconazole	321.0/125.0 (40)	321.0/70.0 (22)
Methacrifos	241.0/209.0 (5)	241.0/124.9 (20)
Methamidophos	142.0/124.9 (10)	142.0/93.9 (10)
Methidathion	302.9/145.0 (2)	302.9/85.0 (10)
Methiocarb	226.1/169.1 (5)	226.1/121.1 (10)
Methiocarb-sulfone	258.0/201.0 (14)	258.0/122.0 (23)
Methiocarb-sulfoxide	242.0/185.0 (14)	242.0/122.1 (28)
Methomyl	163.0/106.0 (5)	163.0/88.0 (5)
Methoxyfenozide	369.1/149.0 (14)	369.1/133.1 (28)
Metobromuron	259.0/169.9 (12)	259.0/148.0 (8)
Metolachlor	284.0/252.1 (8)	284.0/176.1 (20)
Metosulam	418.0/175.0 (25)	418.0/140.0 (25)
Metoxuron	229.0/156.0 (17)	229.0/72.0 (17)
Metribuzin	215.2/187.1 (13)	215.2/84.1 (19)
Metsulfuron-methyl	382.1/199.0 (19)	382.1/167.0 (11)
Mevinphos	225.1/193.0 (1)	225.1/127.0 (11)
Molinate	188.2/126.1 (9)	188.2/55.2 (25)
Monocrotophos	224.2/127.0 (8)	224.2/58.0 (28)
Monolinuron	215.0/148.0 (8)	215.0/125.9 (12)
Monuron	199.1/126.0 (20)	199.1/72.0 (35)
Myclobutanil	289.2/125.1 (27)	289.2/70.2 (15)
Neburon	275.1/88.2 (11)	275.1/57.2 (19)
Nicosulfuron	411.0/213.0 (10)	411.0/182.0 (14)
Nitenpyram	271.1/237.1 (15)	271.1/224.0 (20)
Nuarimol	315.0/252.0 (16)	315.0/81.0 (24)
Omethoate	213.9/182.9 (4)	213.9/125.0 (16)
Orto (2) phenil phenol	170.1/155.0 (22)	170.1/141.0 (34)
Oxadixyl	279.1/219.0 (2)	279.1/102.0 (2)

Oxamyl	237.0/90.0 (0)	237.0/72.0 (10)
Penconazole	284.1/159.0 (29)	284.1/70.0 (13)
Pendimethalin	282.2/212.0 (3)	282.2/194.0 (3)
Phenmedipham	301.0/168.0 (4)	301.0/136.0 (18)
Phenthoate	321.0/246.9 (6)	321.0/163.0 (8)
Phosalone	368.1/322.0 (3)	368.1/182.0 (9)
Phosmet	317.9/160.0 (5)	317.9/133.0 (5)
Phosphamidon	300.0/174.0 (8)	300.0/127.0 (16)
Phoxim	299.0/129.0 (2)	299.0/97.0 (10)
Phthalide	391.3/167.0 (5)	391.3/149.0 (5)
Pirimicarb	239.2/182.1 (11)	239.2/72.1 (15)
Pirimiphos-ethyl	334.2/198.1 (19)	334.2/182.1 (19)
Pirimiphos-methyl	306.2/164.1 (19)	306.2/108.1 (31)
Primisulfuron	469.0/254.0 (4)	469.0/199.0 (6)
Prochloraz	376.1/308.0 (5)	376.1/265.9 (11)
Profenofos	372.9/344.9 (10)	372.9/302.9 (15)
Profoxydim	466.1/280.1 (12)	466.1/180.1 (24)
Prometryn	242.5/200.3 (14)	242.5/68.1 (44)
Propachlor	212.2/170.1 (9)	212.2/94.2 (25)
Propanil	218.0/162.0 (15)	218.0/127.0 (20)
Propaquizafop	444.1/299.1 (20)	444.1/100.1 (15)
Propargite	368.1/231.1 (2)	368.1/175.0 (8)
Propazine	230.2/188.1 (13)	230.2/146.1 (21)
Propham	180.1/138.1 (1)	180.1/120.1 (13)
Propiconazole	342.2/159.0 (27)	342.2/69.2 (17)
Propoxur	210.2/111.1 (7)	210.2/93.1 (21)
Propyzamide	256.1/189.9 (9)	256.1/172.9 (17)
Pymetrozine	218.0/79.0 (16)	218.0/79.0 (50)
Pyrazophos	374.2/222.0 (17)	374.2/194.0 (33)
Pyridaben	365.2/309.1 (7)	365.2/147.1 (23)
Pyridaphenthion	314.2/205.0 (19)	314.2/189.0 (17)
Pyridate	379.2/351.1 (3)	379.2/207.0 (11)
Pyrifenox	297.0/93.0 (15)	297.0/93.0 (35)
Pyrimethanil	200.3/107.0 (11)	200.3/82.0 (23)
Pyriproxyfen	322.2/185.0 (19)	322.2/96.1 (11)
Quizalofop-p	375.1/301.0 (15)	375.1/299.0 (15)
Rimsulfuron	432.1/182.0 (19)	432.1/139.1 (60)
Sethoxydim	328.2/282.2 (10)	328.2/178.1 (15)
Simetryn	214.1/186.1 (0)	214.1/144.1 (2)
Spinosad A	732.6/98.1 (59)	732.6/142.0 (31)
Spinosad D	746.5/98.1 (53)	746.5/142.0 (31)
Spirodiclofen	411.0/313.0 (11)	411.0/71.0 (16)
Spiroxamine	298.2/144.1 (14)	298.2/100.0 (28)
Sulfluramid	526.0/219.0 (7)	526.0/169.0 (17)
Sulfosulfuron	471.0/261.0 (19)	471.0/211.0 (14)
Tebuconazole	308.1/124.9 (36)	308.1/70.0 (18)
Tebufenozide	353.1/297.1 (2)	353.1/133.0 (14)
Tebufenpyrad	334.0/145.0 (28)	334.0/117.0 (40)
Teflubenzuron	379.0/359.0 (2)	379.0/339.0 (2)

Terbutryn	242.2/186.1 (13)	242.2/91.1 (25)
Tetrachlorvinphos	378.9/358,8 (10)	378.9/96.2 (11)
Tetraconazole	372.0/159.0 (35)	372.0/70.0 (20)
Thiabendazole	202.2/175.1 (25)	202.2/131.1 (35)
Thiacloprid	253.0/126.0 (16)	253.0/90.0 (35)
Thiamethoxam	292.1/211.1 (5)	292.1/181.1 (19)
Thiobencarb	258.1/125.1 (25)	258.1/100.1 (5)
Thiodicarb	355.1/108.0 (9)	355.1/88.0 (9)
Tolylfluanid	347.0/238.0 (5)	347.0/137.0 (25)
Tralkoxydim	330.3/284.1 (7)	330.3/138.0 (17)
Triadimefon	294.2/225.1 (7)	294.2/197.1 (11)
Triadimenol	296.1/99.0 (10)	296.1/70.0 (5)
Triallate	304.0/142.8 (22)	304.0/86.0 (10)
Triasulfuron	402.1/167.1 (25)	402.1/140.8 (29)
Trichlorfon	256.8/221.0 (4)	256.8/109.0 (12)
Trifloxystrobin	409.3/206.0 (9)	409.3/186.0 (13)
Triflumizole	346.2/278.0 (3)	346.2/43.2 (21)
Triflumuron	359.0/156.0 (5)	359.0/139.0 (15)
Triticonazole	318.0/125.0 (30)	318.0/70.0 (20)
Tritosulfuron	445.9/195.0 (18)	445.9/145.1 (48)
Oxamyl	237.0/90.0 (0)	237.0/72.0 (10)
Vamidothion	288.1/146.1 (5)	288.1/58.2 (43)
Zoxamide	337.9/188.8 (18)	337.9/186.8 (18)

Table S2. MRM parameters for 52 pesticide residues by GC-MS/MS.

**Pesticide**	**MRM transitions**	
	quantifier ion, *m/z* (collision energy, eV)	quantifier ion, *m/z* (collision energy, eV)
Acetochlor	132.1/117.1 (10)	132.1/131.1 (10)
Alachlor	160.1/131.7 (10)	160.1/188.1 (8)
Aldrin	91.1 /65.0 (15)	91.1 /192.9 (30)
Atrazine	226.3/184.2 (19)	226.3/99.1 (20)
Benfluralin	292.1./264.0 (15)	292.1/160 (10)
Bifenazate	202/117.1 (23)	202/127.1 (28)
Bifenthrin	181.1/160.1 (25)	181.1/166.1 (10)
Bipehynl	153.1/152.1 (15)	154.1/153.1 (15)
Butralin	266/147.1 (24)	266/174.1 (21)
Captafol	79.0/77.0 (14)	79.0/51.0 (20)
Captan	151.0/79.0 (20)	151.0/122 (10)
Carbosulfan	381.3/160.2 (9)	381.3/118.2 (15)
Chlorothalonil	265.9/230.8 (14)	265.9/168.0 (22)
Chlorpropham	213.8/171.8 (2)	213.8/153.8 (12)
Chlorpyrifos-methyl	321.9/289.9 (11)	321.9/125.0 (17)
Cyanophos	243.0/109.0 (12)	243.0/125.0 (14)
Cyhalothrin gamma	229.1/109.0 (15)	229.1/120.1 (10)
Cyhalothrin lambda	197.0/141.0 (12)	197.0/161.0 (12)
Cypermethrin	181.1/152.1 (22)	181.1/127.1 (22)
Dazomet	162.0/89.0 (5)	162.0/46.0 (15)
Deltamethrin	252.9/93.0 (20)	252.9/171.9 (8)

Dichlofluanid	333.1/224.0 (5)	333.1/123.0 (25)
Dicofol	250.0/139.0 (14)	250.0/215.0 (8)
Dieldrin	276.9/241.0 (8)	276.9/170.0 (38)
Dimethipin	118.0/58.0 (6)	118.0/76.0 (6)
Dinobuton	211.0/163.0 (8)	211/117.0 (18)
Disulfoton	275.0/89.0 (5)	275.0/61.0 (20)
Endrin	262.9/193.0 (28)	262.9/191.0 (30)
Esfenvalerate (Fenvalerate)	439.0/169.0 (10)	439.0/167.0 (14)
Fenarimol	330.8/267.9 (20)	330.8/81.1 (32)
Flumetralin	143.0/107.0 (18)	143.0/117.0 (20)
Folpet	259.9/130.0 (14)	259.9/95.0 (20)
Fonofos	247.0/137.0 (5)	247.0/109 (15)
Heptachlor	271.8/236.9 (20)	271.8/117.0 (32)
Iprodione	330.0/287.9 (5)	330.0/245.0 (12)
Metamitron	203.2/104.0 (19)	203.2/42.0 (29)
Nitrapyrin	193.9/133.0 (16)	193.9/157.9 (20)
Oxyfluorfen	362.0/316.0 (10)	362.0/237.1 (25)
Parathion-ethyl	292.0/264.0 (5)	292.0/236.0 (10)
Parathion-metyhl	264.0/231.9 (23)	264.0/124.9 (27)
Permethrin, cis-	183.1/168.1 (14)	183.10/165.1 (10)
Permethrin, trans-	183.1/168.1 (14)	183.10/165.1 (10)
Phorate	261.0/75.0 (10)	261.0/47.0 (28)
Propamocarb	58.0/56.0 (10)	59.0/57.0 (10)
Prothiofos	345.0/268.9 (5)	345.0/240.9 (13)
Pyrimidifen	384.1/282.0 (18)	384.1/328.0 (14)
Simazine	202.1/124.2 (13)	202.1/104.0 (13)
Tau-fluvalinate	250.1/55.0 (20)	250.1/200.0 (20)
Terbufos	288.9/103 (2)	288.9/57.1 (18)
Tetramethrin	332.0/286.0 (10)	332.0/164.0 (29)
Trifluralin	306.1/264.1 (8)	306.1/206.1 (14)
Vinclozolin	285.0/212.0 (12)	285.0/178.0 (14)

Table S3. The in-house validation data for 311 pesticide residues by LC-MS/MS.

Pesticide	Analysed by	LOQ (mg kg⁻¹)	Recovery (%)		Repeatability (%RSD, *n* = 5)		Within-laboratory reproducibility (%RSD, *n* = 10)		*U* (%)
			0.01 mg kg⁻¹	0.05 mg kg⁻¹	0.01 mg kg⁻¹	0.05 mg kg⁻¹	0.01 mg kg⁻¹	0.05 mgkg⁻¹	
2,4-D	LC-MS/MS	0.008	106	110	6.8	3.6	13.2	18.2	44
Acephate	LC-MS/MS	0.008	100	96	19.3	19.1	19.2	13.0	50
Acetamiprid	LC-MS/MS	0.005	91	100	8.4	6.5	8.7	15.1	35
Acibenzolar-s-methyl	LC-MS/MS	0.007	90	84	16.6	14.0	11.9	19.7	49
Aldicarb	LC-MS/MS	0.009	96	106	4.5	4,8	8.8	17.4	39
Aldicarb sulfone	LC-MS/MS	0.005	105	99	7.7	9.5	7.8	13.3	32
Aldicarb sulfoxide	LC-MS/MS	0.007	106	82	3.4	9.1	6.4	19.4	39
Aminocarb	LC-MS/MS	0.006	81	89	9.2	5.6	8.5	6.2	37
Amitraz	LC-MS/MS	0.006	84	97	11.6	5.1	10.1	14.7	42
Anilofos	LC-MS/MS	0.005	84	114	17.5	5.7	14.1	8.5	41
Atrazine-desethyl	LC-MS/MS	0.004	96	99	4.5	7.6	11.4	11.7	36
Atrazine-desisopropyl	LC-MS/MS	0.009	77	96	11.7	9.8	15.1	13.0	43
Azaconazole	LC-MS/MS	0.005	102	102	7.7	3.6	15.1	14.2	41
Azamethiphos	LC-MS/MS	0.006	91	79	16.5	7.5	11.6	14.9	43
Azinphos ethyl	LC-MS/MS	0.007	86	102	10.0	6,6	13.4	10.2	37
Azinphos methyl	LC-MS/MS	0.007	102	101	8.7	6.1	19.2	18.1	46
Azoxystrobin	LC-MS/MS	0.003	82	110	12.5	8.9	13.5	17.8	45
Benalaxyl	LC-MS/MS	0.003	104	99	15.9	3.0	17.8	10.3	40
Benazolin	LC-MS/MS	0.008	97	106	11.8	6.4	16.3	17.5	40
Bendiocarb	LC-MS/MS	0.008	100	103	7.8	11.1	11.8	11.0	34

Benodanil	LC-MS/MS	0.009	97	78	8.2	9.5	12.1	11.2	35
Benoxacor	LC-MS/MS	0.006	83	115	19.5	2.7	18.1	16.7	48
Bensulfuron-methyl	LC-MS/MS	0.006	87	111	15.9	5.4	10.4	13.2	36
Bentazone	LC-MS/MS	0.004	77	106	6.3	9.0	10.4	11.5	41
Bitertanol	LC-MS/MS	0.008	82	89	16.3	4.2	17.1	11.8	41
Bixafen	LC-MS/MS	0.010	91	88	7.5	6.7	11	15.4	34
Boscalid	LC-MS/MS	0.009	108	110	6.7	4.2	17	10.1	37
Bromacil	LC-MS/MS	0.007	102	84	10,5	5.8	18.5	16.2	45
Bromoxynil	LC-MS/MS	0.006	83	112	13.5	5.1	17.5	19.2	49
Bromuconazole	LC-MS/MS	0.007	92	109	8.6	7.2	16.1	15.2	39
Bupirimate	LC-MS/MS	0.004	100	110	3.0	4.9	14.3	11.3	34
Buprofezin	LC-MS/MS	0.004	96	96	5.8	5.2	19.2	11.4	39
Butachlor	LC-MS/MS	0.003	101	99	17.9	8.5	7.0	18.2	38
Butafenacil	LC-MS/MS	0.008	101	97	5.2	5.6	13.1	15.3	39
Butamifos	LC-MS/MS	0.005	100	91	7.7	5.5	13.7	18.6	43
Buturon	LC-MS/MS	0.004	89	103	18.9	9.5	14.7	6.0	40
Butylate	LC-MS/MS	0.006	100	99	6.8	2.8	18.3	14.1	45
Cadusafos	LC-MS/MS	0.005	87	114	17.2	4.5	16.2	4.4	41
Carbaryl	LC-MS/MS	0.004	85	113	15.7	3.7	9.8	15.5	41
Carbendazim	LC-MS/MS	0.006	106	89	13.5	13.3	16.7	11.0	42
Carbofuran	LC-MS/MS	0.005	80	96	11.0	16.8	17.9	16.9	44
Carboxin	LC-MS/MS	0.004	78	105	15.9	8.8	17.1	13.4	44
Chlorbromuron	LC-MS/MS	0.007	84	109	16.5	7.6	18.4	14.1	48
Chlorfluazuron	LC-MS/MS	0.006	82	78	14.9	3.6	14.5	16.2	43
Chloridazon	LC-MS/MS	0.003	98	106	16.1	14.4	17.8	11.5	46

Chloroxuron	LC-MS/MS	0.005	92	114	14.1	3.8	12.9	10.6	43
Chlorpyrifos	LC-MS/MS	0.008	85	112	18.8	7.5	15.4	16.2	46
Chlorsulfuron	LC-MS/MS	0.004	100	102	11.2	18.4	19.0	13.4	49
Cinosulfuron	LC-MS/MS	0.005	96	91	10.4	9.7	10.1	16.4	37
Clethodim	LC-MS/MS	0.004	89	91	15.7	8.3	18.1	9.2	40
Clodinafop propargyl	LC-MS/MS	0.005	89	107	8.0	4.4	12.9	16.9	39
Clofentezine	LC-MS/MS	0.010	96	81	13.8	9.6	18.8	8.3	43
Clomazone	LC-MS/MS	0.004	81	109	17.8	6.4	8.6	8.5	37
Clopyralid	LC-MS/MS	0.009	81	108	16.4	5.3	12.8	12.5	41
Clothianidin	LC-MS/MS	0.009	85	108	8.3	7.5	13.2	11.3	41
Crimidine	LC-MS/MS	0.005	90	113	16.8	6.5	18.1	10.9	43
Crotoxyphos	LC-MS/MS	0.005	90	103	11.5	2.3	13.4	12.1	42
Cyanazine	LC-MS/MS	0.005	86	76	13.4	5.7	11.2	8.6	32
Cyazofamid	LC-MS/MS	0.008	84	110	11.5	5.1	11.6	12.1	41
Cycloate	LC-MS/MS	0.004	82	94	14.9	7.1	14.7	8.4	37
Cyclohexamide	LC-MS/MS	0.009	81	90	14.1	5.7	8.6	12.5	36
Cycloxydim	LC-MS/MS	0.004	83	80	16.9	5.2	13.9	10.6	38
Cycluron	LC-MS/MS	0.004	93	80	11.1	11.2	11.9	18.0	41
Cyflufenamid	LC-MS/MS	0.003	90	98	16.9	7.6	16.6	12.3	44
Cyhalofop-butyl	LC-MS/MS	0.006	83	76	14.1	5.8	17.2	8.1	42
Cymoxanil	LC-MS/MS	0.007	91	97	14.8	9.1	13.6	14.5	39
Cyproconazole	LC-MS/MS	0.010	95	103	11.6	7.2	10.7	10.6	43
Cyprodinil	LC-MS/MS	0.004	82	109	17.8	16.5	11.2	11.1	36
Daimuron	LC-MS/MS	0.004	90	99	9.3	16.1	11.2	12.9	43
Demeton-s-(disulfoton oxon)	LC-MS/MS	0.006	107	92	10.6	7.3	12.2	13.4	43

Demeton-s-methyl sulfone	LC-MS/MS	0.009	76	92	17.1	14.2	10.6	19.3	50
Demeton-s-methyl sulfoxide	LC-MS/MS	0.009	84	107	19.7	9.7	12.4	13.6	44
Desmedipham	LC-MS/MS	0.004	77	111	17.6	5.7	16.8	16.0	50
Desmetryn	LC-MS/MS	0.003	88	79	14.3	9.1	19.4	17.3	48
Diafenthiuron	LC-MS/MS	0.005	85	77	14.0	3.5	12.9	11.1	36
Diazinon	LC-MS/MS	0.003	82	108	11.1	5.9	9.9	14.1	39
Diazinon-oxon	LC-MS/MS	0.004	92	91	18.2	16.3	9.1	13.2	38
Dichlormid	LC-MS/MS	0.004	97	77	8.2	8.9	14.6	19.0	36
Dichlorprop	LC-MS/MS	0.005	78	108	8.2	5.8	18.8	15.8	45
Dichlorvos	LC-MS/MS	0.008	82	94	12.1	8.2	18.9	9.1	41
Diclobutrazol	LC-MS/MS	0.007	90	102	13.9	8.5	13.8	15.9	40
Dicrotophos	LC-MS/MS	0.006	92	92	7.8	7.7	5.5	6.2	19
Diethofencarb	LC-MS/MS	0.005	88	111	17.8	5.4	14.8	11.1	44
Difenoconazole	LC-MS/MS	0.005	89	75	17.5	4.9	17	17.4	44
Difenoxuron	LC-MS/MS	0.003	85	75	15.3	8.2	15.2	17.1	47
Diflubenzuron	LC-MS/MS	0.007	112	93	5.5	9.3	13.5	12.0	33
Dimefuron	LC-MS/MS	0.004	77	113	15.6	6.8	13.3	9.2	35
Dimethachlor	LC-MS/MS	0.003	83	103	17.7	12.9	13.3	9.7	44
Dimethenamid	LC-MS/MS	0.004	78	105	19.4	7.8	10.4	9.7	38
Dimethoate	LC-MS/MS	0.004	92	111	9.8	5.7	15.8	11.3	32
Dimethomorph	LC-MS/MS	0.003	78	96	19.4	3.9	17.5	6.9	46
Dimetilan	LC-MS/MS	0.004	89	101	4.2	13.1	7.5	13.2	32
Dimoxystrobin	LC-MS/MS	0.006	85	113	19	4.1	15.8	12.1	46
Diniconazole	LC-MS/MS	0.007	78	77	12.9	4.9	11.6	16.0	37
Dinitramine	LC-MS/MS	0.009	82	107	6.4	8.7	6.9	15.3	39

Dinocap	LC-MS/MS	0.004	99	93	14.3	5.9	12.0	12.2	37
Dinotefuran	LC-MS/MS	0.011	85	92	12.1	8.3	11.8	9.9	36
Dioxacarb	LC-MS/MS	0.005	80	94	6.0	12.9	18.7	9.7	40
Diphenamid	LC-MS/MS	0.003	78	105	19.4	7.8	10.4	9.7	38
Disulfoton sulfone	LC-MS/MS	0.009	80	116	17.2	4.8	16.8	11.1	41
Disulfoton sulfoxide	LC-MS/MS	0.009	81	109	19.5	7.5	8.9	15.4	45
Dithianon	LC-MS/MS	0.007	82	92	9.4	7.9	18.3	16.7	49
Dithiopyr	LC-MS/MS	0.006	90	102	13.9	8.5	13.8	15.9	40
Diuron	LC-MS/MS	0.004	90	100	19.6	7.9	17.3	12.8	49
Dodine	LC-MS/MS	0.004	87	97	18.4	3.4	14.2	11.2	39
Edifenphos	LC-MS/MS	0.004	83	107	6.4	5.7	10.9	16.1	36
Epoxiconazole	LC-MS/MS	0.004	101	86	11.7	4.4	17.3	13.2	39
Eprinomectin	LC-MS/MS	0.010	85	112	18.6	5.0	19.1	12.8	45
Esprocarb	LC-MS/MS	0.003	86	98	5.3	13.6	14.7	15.8	46
Etaconazole	LC-MS/MS	0.005	85	108	19.6	5.9	15.1	13.9	45
Ethametsulfuron-methyl	LC-MS/MS	0.003	101	101	12.8	9.4	5.4	14.7	44
Ethidimuron	LC-MS/MS	0.004	79	94	14.7	4.8	19.0	10.6	38
Ethiofencarb	LC-MS/MS	0.004	83	104	16.1	6.0	12.3	16.6	45
Ethiofencarb sulfone	LC-MS/MS	0.003	88	83	19.8	9.4	8.7	16.6	42
Ethiofencarb sulfoxide	LC-MS/MS	0.007	99	97	5.8	9.8	10.2	9.2	39
Ethiolate	LC-MS/MS	0.005	85	112	18.6	5.1	19.1	12.8	45
Ethion	LC-MS/MS	0.004	88	88	16.8	4.8	11.8	10.6	35
Ethimirol	LC-MS/MS	0.003	89	112	14.0	8.9	14.2	9.2	45
Ethofumesate	LC-MS/MS	0.007	79	110	13.9	5.5	17.1	17.2	42
Ethoprophos	LC-MS/MS	0.003	80	113	17.3	5.2	13.6	17.2	45

Etofenprox	LC-MS/MS	0.005	84	88	3.3	14.3	15.2	17.0	46
Etoxazole	LC-MS/MS	0.003	84	97	17.9	4.7	18.7	10.2	41
Famoxadone	LC-MS/MS	0.010	97	83	15.5	10.3	16.2	9.8	40
Fenamidone	LC-MS/MS	0.005	88	100	18.3	8.6	16.2	10.8	39
Fenamiphos	LC-MS/MS	0.004	83	108	9.9	8	6.3	18.2	39
Fenamiphos sulfone	LC-MS/MS	0.005	84	99	5.9	9.5	11.9	15.7	42
Fenamiphos sulfoxide	LC-MS/MS	0.005	90	103	14.4	6.2	16.1	11.3	43
Fenazaquin	LC-MS/MS	0.005	80	87	4.6	2.8	19.8	15.5	43
Fenbuconazole	LC-MS/MS	0.008	84	87	14.7	5.2	12.8	19.0	43
Fenhexamid	LC-MS/MS	0.010	85	87	18.1	9.0	13.7	13.2	41
Fenobucarb	LC-MS/MS	0.003	82	109	16.8	13.6	19.1	16.9	49
Fenathiocarb	LC-MS/MS	0.004	98	98	7.1	10.5	12	20.0	43
Fenoxanil	LC-MS/MS	0.007	84	112	12.7	7.2	12.8	19.5	38
Fenoxaprop-ethyl	LC-MS/MS	0.005	83	90	19.4	3.9	19.7	5.2	44
Fenoxycarb	LC-MS/MS	0.004	87	88	15.2	6.5	15.8	11.6	43
Fenpropathrin	LC-MS/MS	0.010	84	102	15.9	7.1	17.9	16.9	45
Fenpropidin	LC-MS/MS	0.004	99	100	10.2	9.6	19.6	16.5	49
Fenpropimorph	LC-MS/MS	0.004	83	92	18.0	5.7	11.2	14.3	38
Fenpyrazamine	LC-MS/MS	0.005	104	77	6.3	4.3	12.3	11.3	34
Fenpyroximate	LC-MS/MS	0.007	92	73	3.9	2.2	16.8	11.9	37
Fensulfothion	LC-MS/MS	0.003	80	105	1.7	16.8	17.2	18.4	48
Fenthion	LC-MS/MS	0.007	86	86	13.7	7.7	19.1	14.3	47
Fenthion oxon	LC-MS/MS	0.003	81	95	6.1	13.4	16.8	17.4	47
Fenthion oxonsulfone	LC-MS/MS	0.004	108	100	6.4	5.1	12.3	13.6	50
Fenthion oxonsulfoxide	LC-MS/MS	0.003	93	97	13.7	12.8	7.7	7.2	36

Fenthion sulfone	LC-MS/MS	0.005	81	115	10.8	6.2	13.9	18.4	44
Fenthion sulfoxide	LC-MS/MS	0.005	88	112	19.3	3.9	13.9	15.4	43
Fenuron	LC-MS/MS	0.003	77	110	18.7	6.9	12.9	13.1	43
Fipronil	LC-MS/MS	0.006	86	82	16.7	9.7	17.4	18.4	50
Flamprop isopropyl	LC-MS/MS	0.003	90	107	16.1	11.5	12.6	14.5	46
Flamprop-methyl	LC-MS/MS	0.004	84	95	16.8	8.8	14.7	13.9	42
Florasulam	LC-MS/MS	0.009	88	93	16.0	4.5	7.8	8.2	35
Fluazifop	LC-MS/MS	0.007	84	89	16.6	14.8	14.8	15.3	42
Fluazifop-p-butyl	LC-MS/MS	0.004	86	91	17.2	4.5	17.6	4.6	49
Fluazinam	LC-MS/MS	0.004	109	113	7.1	4.6	19.3	11.3	38
Flubendiamide	LC-MS/MS	0.006	96	97	13.9	4.7	17.3	14.3	42
Fludioxonil	LC-MS/MS	0.004	87	98	9.0	6.4	11.3	8.4	27
Flufenoxuron	LC-MS/MS	0.008	83	73	15.4	2.1	12.9	8.7	32
Flumetsulam	LC-MS/MS	0.003	96	76	13.9	5.4	7.0	15.9	40
Fluometuron	LC-MS/MS	0.004	88	105	19.7	7.5	13.1	13.1	43
Fluopyram	LC-MS/MS	0.009	90	113	17.4	4.4	11.8	11.9	38
Fluoxastrobin	LC-MS/MS	0.005	83	105	13.0	4.8	10.1	14.6	36
Fluridone	LC-MS/MS	0.004	85	111	16.9	7.7	16.2	14.1	47
Flurochloridone	LC-MS/MS	0.009	96	103	13.8	6.9	13.3	10.1	38
Flutolanil	LC-MS/MS	0.004	81	114	19.2	4.7	15.3	15.4	42
Flutriafol	LC-MS/MS	0.006	93	83	16.9	7.7	13.6	17.1	46
Fluxapyroxad	LC-MS/MS	0.004	87	114	18.2	4.9	12.3	14.7	44
Fomesafen	LC-MS/MS	0.008	79	95	13.7	7.4	14.9	13.0	39
Forchlorfenuron	LC-MS/MS	0.007	98	85	13.9	7.1	8.9	7.8	39
Formothion	LC-MS/MS	0.006	97	108	18.8	13.8	18.1	11.9	44

Furalaxyl	LC-MS/MS	0.004	104	100	12.7	5.4	10.4	8.6	34
Furathiocarb	LC-MS/MS	0.004	104	99	15.9	4.7	14.3	17.8	43
Haloxyfop-etotyl	LC-MS/MS	0.004	100	96	16.3	13.5	12.1	11.3	40
Haloxyfop-methyl	LC-MS/MS	0.004	108	84	6.2	7.3	13.1	10.9	32
Heptenophos	LC-MS/MS	0.005	98	101	6.6	15.6	9.6	9.4	35
Hexaconazole	LC-MS/MS	0.009	101	96	7.1	11.4	13.7	7.9	37
Hexaflumuron	LC-MS/MS	0.006	102	90	9.2	10.4	13.0	7.9	35
Hexazinone	LC-MS/MS	0.004	108	107	14.0	5.8	17.3	13.4	46
Hexythiazox	LC-MS/MS	0.006	87	80	17.0	8.4	14.5	8.5	39
Imazalil	LC-MS/MS	0.006	95	99	4.6	6.5	12.3	14.9	37
Imazapyr	LC-MS/MS	0.010	83	99	11.5	9.0	15.2	12.0	37
Imazaquin	LC-MS/MS	0.004	102	107	13.7	8.5	19.4	14.5	47
Imibenconazole	LC-MS/MS	0.008	102	107	8.8	4.6	9.3	17.8	31
Imidacloprid	LC-MS/MS	0.007	102	106	11.0	2.6	15.6	17.8	45
Indanofan	LC-MS/MS	0.009	113	108	2.8	5.9	16.1	14.5	38
Indoxacarb	LC-MS/MS	0.007	104	93	15.4	13.6	17.5	19.9	49
Iodosulfuron-methyl sodium	LC-MS/MS	0.007	93	93	13.0	13.5	12.0	13.1	34
Ioxynil	LC-MS/MS	0.008	80	106	8.2	3.8	15.8	16.5	41
Ipconazole	LC-MS/MS	0.005	102	84	9.2	4.8	15.8	16.6	45
Iprobenfos	LC-MS/MS	0.007	98	97	15.4	19.6	11.3	15.5	45
Isocarbamid	LC-MS/MS	0.004	107	97	6.9	4.3	15.8	19.3	44
Isoprocarb	LC-MS/MS	0.005	99	104	14.2	14.1	11.9	18.2	45
Isopropalin	LC-MS/MS	0.006	100	101	6.0	6.9	13.9	12.8	39
Isopyrazam	LC-MS/MS	0.004	98	103	14.3	2.6	13.1	12.7	33
Isoxaben	LC-MS/MS	0.003	108	97	4.9	4.7	16.3	5.0	37

Isoxathion	LC-MS/MS	0.004	106	100	8.0	6.5	10.8	15.6	36
Kresoxim-methyl	LC-MS/MS	0.005	106	92	15.7	14.9	10.6	17.0	46
Lactofen	LC-MS/MS	0.007	100	111	10.0	9.7	15.1	12.8	41
Lenacil	LC-MS/MS	0.004	104	108	5.2	3.6	8.5	16.7	35
Linuron	LC-MS/MS	0.005	97	92	11.8	8.8	16.1	17.1	47
Lufenuron	LC-MS/MS	0.007	91	102	7.0	9.3	7.5	16.7	45
Malaoxon	LC-MS/MS	0.004	106	107	3.1	3.2	6.4	18.3	39
Malathion	LC-MS/MS	0.005	89	100	19.1	15.2	12.0	19.9	48
MCPA	LC-MS/MS	0.008	95	103	8.3	16.6	12.2	17.4	45
Mecarbam	LC-MS/MS	0.003	98	105	18.7	8.3	17.6	8.1	45
Mefenacet	LC-MS/MS	0.007	99	104	5.2	3.0	19.9	16.1	47
Mepanipyrim	LC-MS/MS	0.004	106	105	9.0	7.5	16.8	19.1	50
Mepronil	LC-MS/MS	0.004	105	94	8.4	4.8	6.2	15.0	36
Mesosulfuron-methyl	LC-MS/MS	0.008	98	95	13.7	6.4	14.9	14.8	39
Metaflumizone	LC-MS/MS	0.009	101	105	6.6	10.3	14.0	13.7	44
Metalaxyl-M	LC-MS/MS	0.004	114	112	14.1	11.7	17.7	10.5	44
Metconazole	LC-MS/MS	0.004	108	106	4.4	6.0	11.6	4.7	34
Methacrifos	LC-MS/MS	0.006	104	110	10.8	11.0	14.3	17.8	46
Methamidophos	LC-MS/MS	0.007	97	103	17.5	13.1	10.3	15.6	42
Methidathion	LC-MS/MS	0.005	94	95	8.9	12.3	17.5	14.3	40
Methiocarb	LC-MS/MS	0.005	112	118	4.5	6.1	13.6	18.5	37
Methiocarb-sulfone	LC-MS/MS	0.004	115	109	14.9	9.3	13.6	13.9	39
Methiocarb-sulfoxide	LC-MS/MS	0.005	105	97	13.6	7.6	17.8	13.9	41
Methomyl	LC-MS/MS	0.007	98	94	4.7	19.5	7.4	17.9	42
Methoxyfenozide	LC-MS/MS	0.005	90	100	17.7	15.4	17.0	7.7	46

Metobromuron	LC-MS/MS	0.005	106	111	15.0	5.0	17.7	17.6	48
Metolachlor	LC-MS/MS	0.007	101	93	6.3	10.0	17.7	11.6	37
Metosulam	LC-MS/MS	0.005	109	108	3.8	13.9	18.4	11.1	41
Metoxuron	LC-MS/MS	0.004	100	103	5.3	5.1	18.7	16.1	46
Metribuzin	LC-MS/MS	0.009	107	107	9.2	8.7	12.0	14.6	39
Metsulfuron-methyl	LC-MS/MS	0.005	109	98	6.0	5.9	11.2	6.1	36
Mevinphos	LC-MS/MS	0.005	113	109	7.8	7.8	10.0	12.0	33
Molinate	LC-MS/MS	0.006	113	93	4.2	4.5	11.4	6.1	32
Monocrotophos	LC-MS/MS	0.009	101	93	6.6	10.7	7.6	16.4	46
Monolinuron	LC-MS/MS	0.005	106	110	8.1	3.0	7.6	19.1	42
Monuron	LC-MS/MS	0.004	114	102	4.8	7.0	13.1	11.6	41
Myclobutanil	LC-MS/MS	0.005	105	113	8.2	2.8	18.8	11.6	40
Neburon	LC-MS/MS	0.008	101	94	12.4	4.4	14.7	18.1	44
Nicosulfuron	LC-MS/MS	0.004	109	110	5.2	4.7	10.2	5.6	33
Nitenpyram	LC-MS/MS	0.009	92	94	17.8	10.1	3.9	17.3	40
Nuarimol	LC-MS/MS	0.004	93	91	16.0	7.4	15.2	8.9	41
Omethoate	LC-MS/MS	0.010	76	94	8.8	9.4	13.9	7.9	45
Orto (2) phenil phenol	LC-MS/MS	0.009	110	110	8.4	6.2	8.6	7.9	37
Oxadixyl	LC-MS/MS	0.010	110	109	4.4	7.3	17.0	17.3	40
Oxamyl	LC-MS/MS	0.008	110	110	12.7	17.5	12.0	17.8	46
Penconazole	LC-MS/MS	0.004	109	95	10.6	3.4	8.9	12.8	40
Pendimethalin	LC-MS/MS	0.007	106	104	8.7	12.0	10.1	12.1	34
Phenmedipham	LC-MS/MS	0.005	116	100	1.4	6.1	11.2	19.2	40
Phenthoate	LC-MS/MS	0.005	110	103	3.8	5.0	14.2	10.0	34
Phosalone	LC-MS/MS	0.008	113	112	2.6	5.4	9.9	18.0	37

Phosmet	LC-MS/MS	0.005	95	112	3.7	3.7	12.6	15.8	36
Phosphamidon	LC-MS/MS	0.004	109	112	5.9	4.8	6.2	15.8	34
Phoxim	LC-MS/MS	0.004	104	94	9.4	13.7	17.7	14.3	45
Phthalide	LC-MS/MS	0.009	116	106	3.2	7.3	12.4	7.7	36
Pirimicarb	LC-MS/MS	0.005	113	109	5.7	8.3	14.3	11.7	40
Pirimiphos-ethyl	LC-MS/MS	0.005	106	99	5.8	5.4	17.5	18.4	42
Pirimiphos-methyl	LC-MS/MS	0.005	105	102	8.3	8.7	11.8	19.3	44
Primisulfuron	LC-MS/MS	0.008	103	109	2.5	3.6	18.0	18.9	48
Prochloraz	LC-MS/MS	0.006	116	112	3.7	4.9	15.7	9.8	40
Profenofos	LC-MS/MS	0.004	112	113	11.8	3.8	18.0	12.2	39
Profoxydim	LC-MS/MS	0.004	95	98	13.8	17.9	12.4	7.7	36
Prometryn	LC-MS/MS	0.005	110	89	7.3	4.3	10.1	11.3	39
Propachlor	LC-MS/MS	0.003	110	100	6.6	2.9	4.5	17.2	32
Propanil	LC-MS/MS	0.009	84	108	9.6	6.3	16.2	10.4	37
Propaquizafop	LC-MS/MS	0.007	103	111	12.1	7.8	17.7	13.3	40
Propargite	LC-MS/MS	0.004	92	96	16.8	12.7	9.2	10.0	36
Propazine	LC-MS/MS	0.006	104	94	6.6	3.7	19.8	19.4	47
Propham	LC-MS/MS	0.010	107	101	10.4	13.2	8.3	9.9	34
Propiconazole	LC-MS/MS	0.005	101	111	6.7	5.7	14.0	12.2	35
Propoxur	LC-MS/MS	0.009	108	106	9.4	4.6	16.4	16.1	42
Propyzamide	LC-MS/MS	0.007	113	101	4.2	14.0	16.7	11.2	45
Pymetrozine	LC-MS/MS	0.008	99	95	7.4	9.8	8.5	18.7	41
Pyrazophos	LC-MS/MS	0.006	100	109	14.7	9.6	13.6	12.7	41
Pyridaben	LC-MS/MS	0.005	104	107	8.4	5.9	10.3	14.7	35
Pyridaphenthion	LC-MS/MS	0.006	106	96	3.5	10.6	9.1	14.1	38

Pyridate	LC-MS/MS	0.006	86	87	4.4	5.4	13.6	14.2	38
Pyrifenox	LC-MS/MS	0.004	105	85	11.7	9.8	5.1	15.1	35
Pyrimethanil	LC-MS/MS	0.004	113	103	4.5	3.0	17.4	19.6	43
Pyriproxyfen	LC-MS/MS	0.004	107	99	6.6	6.7	18.7	9.1	39
Quizalofop-p	LC-MS/MS	0.010	103	111	12.1	7.8	17.7	13.3	40
Rimsulfuron	LC-MS/MS	0.004	106	101	7.6	8.9	9.7	10.9	36
Sethoxydim	LC-MS/MS	0.005	111	103	4.7	6.1	18.2	13.2	41
Simetryn	LC-MS/MS	0.004	107	104	3.0	5.0	16.5	14.2	39
Spinosad A	LC-MS/MS	0.009	77	99	10.8	6.8	13.4	12.9	40
Spinosad D	LC-MS/MS	0.004	93	90	10.3	5.1	13.0	19.1	42
Spirodiclofen	LC-MS/MS	0.009	113	98	5.1	14.9	6.3	19.7	40
Spiroxamine	LC-MS/MS	0.003	109	102	6.1	10.6	9.8	10.6	35
Sulfluramid	LC-MS/MS	0.005	105	96	5.5	4.3	7.8	13.7	34
Sulfosulfuron	LC-MS/MS	0.006	99	100	8.8	5.9	7.9	15.6	38
Tebuconazole	LC-MS/MS	0.006	94	95	12.9	9.2	17.0	11.5	43
Tebufenozide	LC-MS/MS	0.004	99	103	19.1	15.6	18.8	10.0	46
Tebufenpyrad	LC-MS/MS	0.005	107	107	5.7	8.4	15.1	8.1	31
Teflubenzuron	LC-MS/MS	0.006	105	102	8.5	10.6	13.7	11.2	38
Terbutryn	LC-MS/MS	0.004	100	93	5.5	4.6	10.7	19.1	43
Tetrachlorvinphos	LC-MS/MS	0.010	105	99	10.9	6.1	8.1	13.9	37
Tetraconazole	LC-MS/MS	0.006	106	102	8.1	9.3	6.6	9.2	33
Thiabendazole	LC-MS/MS	0.005	83	101	10.7	5.2	15.0	9.2	38
Thiacloprid	LC-MS/MS	0.004	106	103	7.8	6.1	12.7	16.5	39
Thiamethoxam	LC-MS/MS	0.009	94	93	12.8	8.6	14.5	16.8	44
Thiobencarb	LC-MS/MS	0.005	96	94	11.8	10.1	11.5	15.9	44

Thiodicarb	LC-MS/MS	0.008	108	106	9.4	10.5	14.7	14.1	38
Tolylfluanid	LC-MS/MS	0.006	106	105	5.9	14.0	12.5	12.0	45
Tralkoxydim	LC-MS/MS	0.006	105	102	6.1	5.9	17.4	13.4	38
Triadimefon	LC-MS/MS	0.005	104	101	10.1	6.6	8.2	11.9	34
Triadimenol	LC-MS/MS	0.008	103	97	9.2	9.4	4.4	9.6	27
Triallate	LC-MS/MS	0.009	106	103	9.4	9.7	15.4	18.1	46
Triasulfuron	LC-MS/MS	0.008	109	104	6.2	7.7	7.8	9.1	34
Trichlorfon	LC-MS/MS	0.008	103	86	11.9	7.6	13.7	16.3	45
Trifloxystrobin	LC-MS/MS	0.005	106	99	6.0	10.7	10.5	11.7	37
Triflumizole	LC-MS/MS	0.006	99	101	14.3	10.2	15.1	12.7	44
Triflumuron	LC-MS/MS	0.008	108	109	7.3	3.1	7.8	5.8	22
Triticonazole	LC-MS/MS	0.005	104	109	7.4	4.3	13.6	6.1	37
Tritosulfuron	LC-MS/MS	0.009	100	95	11.4	15.2	17.1	16.1	50
Oaxamyl	LC-MS/MS	0.009	83	111	12.8	2.1	13.7	9.4	37
Vamidothion	LC-MS/MS	0.007	106	108	7.4	5.2	6.3	7.4	32
Zoxamide	LC-MS/MS	0.005	112	104	1.5	3.2	14.3	15.9	39

Table S4. The in-house validation data for 52 pesticide residues by GC-MS/MS.

**Pesticide**	**Analysed by**	**LOQ (mg kg^{-1})**	**Recovery (%)**		**Repeatability (%RSD, *n* = 5)**		**Within-laboratory reproducibility (%RSD, *n* = 10)**		***U* (%)**
			0.01 mg kg^{-1}	0.05 mg kg^{-1}	0.01 mg kg^{-1}	0.05 mg kg^{-1}	0.01 mg kg^{-1}	0.05 mgkg^{-1}	
Acetochlor	GC-MS/MS	0.011	80	105	7.8	6.7	10.5	18.6	42
Alachlor	GC-MS/MS	0.011	92	100	8.5	10.1	12.8	16.2	38
Aldrin	GC-MS/MS	0.011	85	81	7.5	6.4	6.0	10.8	25
Atrazine	GC-MS/MS	0.012	81	112	9.8	19.0	13.1	13.7	43
Benfluralin	GC-MS/MS	0.009	109	85	7.3	5.5	12.7	12.3	29

Bifenazate	GC-MS/MS	0.009	82	108	19.8	5.4	11.5	15.4	45
Bifenthrin	GC-MS/MS	0.011	105	82	14.5	6.0	17.6	19.6	49
Bipehynl	GC-MS/MS	0.010	97	78	3.6	6.9	6.3	10.2	20
Butralin	GC-MS/MS	0.009	95	87	11.3	18.7	14.6	15.7	42
Captafol	GC-MS/MS	0.010	106	89	13.5	13.3	16.7	11.2	42
Captan	GC-MS/MS	0.010	105	98	6.1	11.8	5.3	14.6	30
Carbosulfan	GC-MS/MS	0.009	98	86	19.6	15.5	18.6	15.4	49
Chlorothalonil	GC-MS/MS	0.010	105	93	6.3	15.2	6.5	8.8	26
Chlorpropham	GC-MS/MS	0.010	96	98	13.4	7.3	15.1	6.3	37
Chlorpyrifos-methyl	GC-MS/MS	0.010	79	95	10.1	8.0	19.2	5.2	36
Cyanophos	GC-MS/MS	0.009	86	85	15.9	11.2	13.8	14.9	39
Cyhalothrin gamma	GC-MS/MS	0.008	93	78	15.5	8.4	18.1	3.8	43
Cyhalothrin lambda	GC-MS/MS	0.009	109	113	8.3	3.8	19.7	4.7	45
Cypermethrin	GC-MS/MS	0.009	96	75	13.1	4.1	18.3	14.3	48
Dazomet	GC-MS/MS	0.012	91	81	10.4	12.3	14.1	11.0	36
Deltamethrin	GC-MS/MS	0.012	85	75	18.3	3.1	12.4	14.5	44
Dichlofluanid	GC-MS/MS	0.009	78	107	6.1	6.2	15.4	19.6	41
Dicofol	GC-MS/MS	0.009	80	83	5.9	5.2	8.1	6.7	22
Dieldrin	GC-MS/MS	0.009	91	87	5.4	8.5	3.4	10.8	27
Dimethipin	GC-MS/MS	0.009	87	111	15.7	5.1	9.9	13.9	35
Dinobuton	GC-MS/MS	0.011	99	77	15.4	7.7	16.1	11.2	32
Disulfoton	GC-MS/MS	0.010	80	82	17.9	7.9	9.0	13.2	40
Endrin	GC-MS/MS	0.013	106	100	6.2	9.2	16.7	3.5	34
Esfenvalerate (Fenvalerate)	GC-MS/MS	0.011	105	79	11.8	5.4	12.8	14.2	36
Fenarimol	GC-MS/MS	0.011	86	88	13	5.7	18.9	16.5	48

Flumetralin	GC-MS/MS	0.011	104	90	18.3	6.7	17.6	17.3	41
Folpet	GC-MS/MS	0.009	93	90	12.1	15.1	8.8	10.7	34
Fonofos	GC-MS/MS	0.010	83	78	8.6	5.7	15.9	15.5	40
Heptachlor	GC-MS/MS	0.009	91	87	12.8	12.6	14.9	12.3	37
Iprodione	GC-MS/MS	0.011	103	111	15.7	5.4	18.4	17.2	48
Metamitron	GC-MS/MS	0.012	101	94	7.2	18.8	18.2	12.3	45
Nitrapyrin	GC-MS/MS	0.010	105	105	8.5	3.2	16.3	17.3	44
Oxyfluorfen	GC-MS/MS	0.008	95	86	13.3	19.3	12.4	5.1	36
Parathion-ethyl	GC-MS/MS	0.010	104	97	14.6	10.4	11.7	6.7	38
Parathion-metyhl	GC-MS/MS	0.011	96	91	12.9	12.4	5.1	12.4	33
Permethrin, cis-	GC-MS/MS	0.010	100	103	13.3	9.0	18.1	11.0	45
Permethrin, trans-	GC-MS/MS	0.010	106	92	9.6	6.3	18.0	14.1	46
Phorate	GC-MS/MS	0.009	107	96	7.7	10.8	5.6	12.3	32
Propamocarb	GC-MS/MS	0.011	86	80	10.2	4.8	5.7	12.6	32
Prothiofos	GC-MS/MS	0.010	94	93	19.2	9.4	19.7	11.9	49
Pyrimidifen	GC-MS/MS	0.011	117	87	3.2	16.7	13.2	13.5	37
Simazine	GC-MS/MS	0.011	105	114	14.1	3.6	6.4	19.5	40
Tau-fluvalinate	GC-MS/MS	0.010	93	107	13.7	12.0	6.8	15.1	46
Terbufos	GC-MS/MS	0.008	86	91	16.7	17.8	17.3	8.3	44
Tetramethrin	GC-MS/MS	0.010	116	109	4.1	5.4	18.3	12.0	41
Trifluralin	GC-MS/MS	0.010	108	102	4.8	9.0	8.2	16.1	34
Vinclozolin	GC-MS/MS	0.010	101	107	17.5	7.0	12.6	9.5	35
